# Application of Donabedian Three-Dimensional Model in Outpatient Care Quality: A Scoping Review

**DOI:** 10.1155/jonm/6893336

**Published:** 2025-04-24

**Authors:** Jinrong Yang, Fan Liu, Chunxia Yang, Jingyi Wei, Yonghong Ma, Lisheng Xu, Jingying Xie, Jingjun Wang

**Affiliations:** ^1^Department of Periodontics, State Key Laboratory of Oral Diseases, National Center for Stomatology, National Clinical Research Center for Oral Diseases, West China Hospital of Stomatology, Sichuan University, Chengdu 610041, Sichuan, China; ^2^Nursing Department, State Key Laboratory of Oral Diseases, National Center for Stomatology, National Clinical Research Center for Oral Diseases, West China Hospital of Stomatology, Sichuan University, Chengdu 610041, Sichuan, China; ^3^Department of Central Sterile Supply, State Key Laboratory of Oral Diseases, National Center for Stomatology, National Clinical Research Center for Oral Diseases, West China Hospital of Stomatology, Sichuan University, Chengdu 610041, Sichuan, China; ^4^Department of Oral Implantology, State Key Laboratory of Oral Diseases, National Center for Stomatology, National Clinical Research Center for Oral Diseases, West China Hospital of Stomatology, Sichuan University, Chengdu 610041, Sichuan, China; ^5^Department of Prosthodontics, State Key Laboratory of Oral Diseases, National Center for Stomatology, National Clinical Research Center for Oral Diseases, West China Hospital of Stomatology, Sichuan University, Chengdu 610041, Sichuan, China; ^6^Department of Trauma and Plastic Surgery, State Key Laboratory of Oral Diseases, National Center for Stomatology, National Clinical Research Center for Oral Diseases, West China Hospital of Stomatology, Sichuan University, Chengdu 610041, Sichuan, China; ^7^Department of Head and Neck Oncology Surgery, State Key Laboratory of Oral Diseases, National Center for Stomatology, National Clinical Research Center for Oral Diseases, West China Hospital of Stomatology, Sichuan University, Chengdu 610041, Sichuan, China

## Abstract

**Background:** The mobility of outpatients, frequent treatment sessions, and varying illness severity challenge nursing care and hinder quality improvement efforts. The Donabedian model effectively evaluates nursing quality and is widely used in operating rooms, intensive care units and oncology wards. However, its application in outpatient nursing remains unclear.

**Aim:** To overview the Donabedian model's application in outpatient care quality.

**Methods:** A scoping review was conducted using the Arksey and O'Malley framework, adhering to PRISMA-ScR. Searches for eligible papers were performed on PubMed, OVID, Web of Science, Embase, CNKI, CBM, and Wan Fang in June 2024. Data were analyzed narratively using an inductive approach, with visual mapping done through ArcGIS and Origin.

**Results:** A total of 18 studies were included in the review. Literature review, Delphi and semistructured interview were widely used in the mixed method studies. The primary settings involved dental clinics and health examination centers, followed by traditional Chinese medicine clinics and pediatric clinics. The studies spanned various regions: 2 in Canada, 3 in Germany, and 13 in China. Structural indicators evaluate outpatient care systems and resources, while process indicators emphasize specialty nursing practice, safety, infection control, and humanistic care. Satisfaction, adverse events, and quality care serve as key secondary indicators for outcome assessment.

**Conclusions:** The research on outpatient care quality based on the Donabedian model is primarily mixed, with a need to expand interventional and qualitative studies. The structure-process-outcome quality indicators have varying focuses that require further verification and coordination. Structural indicators offer more policy and resource support, while process indicators differ across outpatient specialties, leading to heterogeneous specialist nursing practice metrics. Satisfaction and adverse events are crucial components in evaluating outpatient nursing quality outcomes.

**Implications for Nursing Management:** The application of the Donabedian model offers care managers practical ideas and references for outpatient care quality improvement.

## 1. Introduction

Nursing quality is a crucial component of medical quality and serves as a key evaluation metric for assessing the service quality of healthcare institutions [1–3]. Managers, nurses, and patients are the three key components in the examination of quality of care. Managers often consider the nursing system, service costs, resource supply, and nursing complaints when making decisions. Nurses, as the central aspect of quality control, primarily define the quality of care in terms of professional skills and outcomes. Patients, in turn, evaluate the quality of care based on the timeliness, effectiveness, and their expectations regarding the delivery of care [4]. Coulon et al. [5] utilized qualitative research methodology to propose that the four key themes of quality care are professional competence, humanistic care, practicality, and humanitarianism. According to Kunaviktikul et al. [6]; the quality of care was contingent upon meeting patients' physical, psychological, and other needs. The Joint Commission International (JCI) expands the concept of quality care to encompass nursing policies, facility and equipment audits, process improvement initiatives, and patient safety measures [7]. Additionally, the World Health Organization (WHO) emphasizes that quality care should address effectiveness, safety, and person-centeredness while meeting standards for timeliness, equity, integration, and efficiency. As the medical paradigm shifts and modern nursing continues to evolve, there is an ongoing refinement of the definition of quality care. In 2022, Chinese scholars Shi and Xi [8] proposed that nursing quality is a broad, abstract concept that should not be limited to the effectiveness of basic nursing services or the level of technology, but should simultaneously cover a variety of topics such as professional nursing, humanized nursing, patient safety, nursing effectiveness, and patient needs. This suggests that the quality of care is not only determined by whether care services meet standard requirements, but also by the extent to which the explicit or implicit needs of care recipients are fulfilled. Therefore, the quality of nursing care includes technical and service attributes that directly respond to the nature and characteristics of nursing care, and is a synthesis of the effectiveness of nursing staff in providing patients with specialized and basic nursing services and in meeting the reasonable needs of patients. The enhancement of nursing quality plays a critical role in improving the safety and effectiveness of nursing services [4, 9, 10], as well as in promoting optimal health outcomes for individuals and populations.

The three-dimensional quality structure model was proposed by American scholar Avedis Donabedian in 1966 as a theory that defines quality attributes in terms of structure, process, and outcome [11, 12]. Through the exploration and active application of scholars from various countries, the theoretical connotation of Donabedian model has been deepened, and it has become a globally recognized, scientific and practical model of nursing quality. Structural quality is the organizational element that guarantees the implementation of nursing activities [13]. Its scope encompasses the evaluation of nursing facilities, human resources and organizational systems [14]. The researcher [15] pointed out that structural indicators were the basis for supporting successful nursing care. Process quality is the evaluation of nursing practice, aimed at ensuring that the nursing staff carries out standardized nursing operations, provides health education, and delivers humanistic care to patients during practice. It focuses on process control to ensure nursing practice. Its scope of evaluation includes the assessment of the feasibility of implementing nursing activities and the fulfillment of nursing practice standards [16, 17]. Outcome quality is the evaluation of the final effect of nursing activities, which includes physical, psychological, and health-related behaviors. Outcome quality is directly influenced by structural and process quality [18]. Structural and process evaluations are designed to ensure that nursing behaviors are practiced in an optimal environment with standard measures. Outcome evaluation is a reflection of the effectiveness of structural and process controls. The Donabedian model has been widely used in nursing practice in clinical departments such as emergency departments, operating rooms, intensive care units, and oncology wards [19–21]. The application mode, type and sensitive indicators have been extensively explored [22, 23]. In addition to this, a series of quality-of-care indicators have been studied for chronic diseases including heart failure, depression, cirrhosis, diabetes, and chronic obstructive pulmonary disease [24–26]. In contrast, utilization in outpatient settings is rarely reported.

Outpatient clinic as the first part of the patient to receive treatment, is the window of the hospital's image [27]. The quality of outpatient nursing services directly reflects the hospital's overall reputation and social impact [28]. Compared to inpatients, outpatients exhibit greater mobility, shorter diagnosis and treatment times, a higher number of diagnosis and treatment procedures, as well as varying degrees of illness severity [29]. Outpatient nurses are often faced with the complexities of interpersonal relationships and the challenges of dealing with the uneven distribution of healthcare resources [30]. At the same time, due to changes in people's lifestyles, work pressure has significantly increased [31], and irregular eating and sleeping patterns have become the norm [32]. This has led to a higher incidence of many diseases, consequently increasing the rate of outpatient visits. All of these unfavorable factors and weaknesses present many challenges to improving the quality of care. Poor quality of care services often affects the patient experience and even leads to medical disputes [4]. Furthermore, the shift in the medical model has led to an increase in people's expectations for the quality of outpatient care [30]. As a result, enhancing the quality of outpatient care has gradually emerged as one of the key medical issues that needs to be addressed [33]. The nursing concept of “people-oriented, patient-centered” guides outpatient nursing staff to provide medical services at physiological, social, and psychological levels in order to ensure the quality of outpatient care. In conclusion, considering the unique nature of the outpatient setting, this study has summarized the application of the Donabedian model in assessing the quality of outpatient nursing. Given the extensive nature of this research topic and our anticipation for a more visual and personalized presentation of findings, we conducted a scoping review to inform researchers about the current developments and status within the field. This provides a valuable reference and foundation for enhancing the delivery of high-quality outpatient nursing care.

## 2. Aim and Research Questions

This scoping review aimed to provide an overview of the application of the Donabedian model in outpatient care quality.

**Figure 1:**
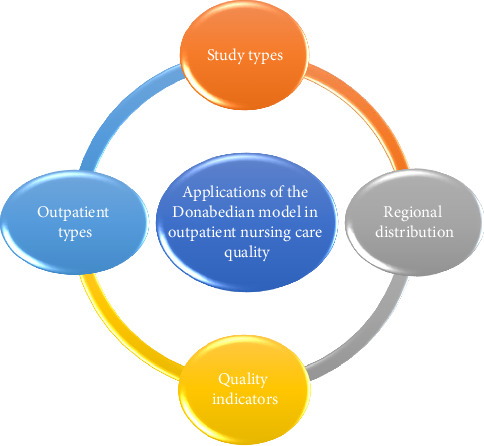
Four specific points of the research questions.

This scoping review addressed two questions including 4 specific points (see Figure 1):

1. How has the Donabedian model been used in outpatient care quality research including study types, outpatient types and regional distribution?
2. What are the quality indicators of outpatient care according to the Donabedian model (structure-process-outcome)?

## 3. Methods

### 3.1. Design

The scoping review is a research method used to identify the literature on a specific topic or research area, thereby providing key concepts, research gaps, evidence sources, and types of guidance for clinical practice, policy development, and research conduct [34]. Arksey and O'Malley [35] five steps of a scoping review were used to guide this report including (1) defining the research question; (2) locating and identifying relevant studies; (3) selecting relevant studies; (4) graphing the data; and (5) collating, summarizing and reporting the results. The Preferred Reporting Items for Systematic reviews and Meta-Analyses Extension for Scoping Reviews (PRISMA-ScR) was used to optimize reporting and improve fidelity [36]. This review was registered with OSF (https://doi.org/10.17605/OSF.IO/Z65FW).

### 3.2. Eligibility Criteria

We used the PCC framework to define the study eligibility criteria. Population: the Donabedian model; Concept: nursing care quality; Context: in outpatient diagnosis and treatment. This scoping review encompassed quantitative, qualitative, or mixed studies published in peer-reviewed academic journals between January 2019 and June 2024. Studies utilizing the Donabedian model to facilitate assessment of outpatient care quality or as an intervention strategy to augment outpatient care quality were included. Research protocols, letters to the editor, reviews, and articles lacking full text availability were excluded from consideration.

### 3.3. Information Sources and Search Strategy

In June 2024, a systematic search was conducted across multiple databases including PubMed, OVID, Web of Science, Embase, CNKI, CBM, and Wan Fang to identify eligible papers. To maximize sensitivity, specific terms were strategically integrated for each individual database. The comprehensive search strategy encompassing all the included search terms utilized for each respective database was shown in the Supporting Information (Table S1). Furthermore, a manual screening of the reference lists of included publications was also undertaken.

### 3.4. Study Selection

An independent researcher conducted an electronic database search and imported all citation lists and abstracts into the Endnote X7.7 software for analysis. The research screening process is divided into title and abstract screening, as well as full-text screening. Prior to the formal screening process, the project manager (the corresponding author) provided comprehensive training for researchers in order to ensure consistency in screening and minimize selection bias. Two independent reviewers removed all duplicates and then independently screened the remaining titles and abstracts to determine which met all inclusion criteria. At least two out of the eight researchers independently assessed the remaining full-text publications based on the inclusion and exclusion criteria. Any discrepancies in the selection process were resolved through discussion, ultimately reaching a consensus on each study.

### 3.5. Data Extraction and Charting

The data extraction process was an iterative process involving regular discussions among all authors. Two authors conducted the data extraction according to the predetermined criteria (e.g., authors' names and year of publication, country, objectives, ambulatory institution, research methodology, and key findings). The other three authors then carefully examine the extracted articles to identify any missing information, with the goal of collecting all relevant study characteristics. In the event of disagreements regarding review information, the final author also participated in the discussion until a consensus was reached. To ensure rigor and authenticity, the final data extraction content was reviewed and determined by all authors. The results were analyzed and two additional researchers utilized ArcGIS and Origin software to generate regional distribution maps and 3D pie charts in order to present data information more intuitively and address review questions effectively.

### 3.6. Date Synthesis and Presentation

After extracting and analyzing the data, a narrative synthesis was used to summarize [27] and the main findings were presented through both descriptive text and tabular format: the initial step involves two researchers systematically identifying all free code/variables mentioned in the article, line by line. Secondly, incorporate the free code into the primary theme. The third step involves deriving the secondary theme following a comparative analysis of the main theme. Finally, the research team has integrated the Donabedian model into their assessment of outpatient care quality. This includes a detailed description of the application used, the regional distribution of the study, specific outpatient institutions involved, and sensitive indicators utilized to measure ambulatory care quality.

## 4. Results

### 4.1. Search Results

**Figure 2:**
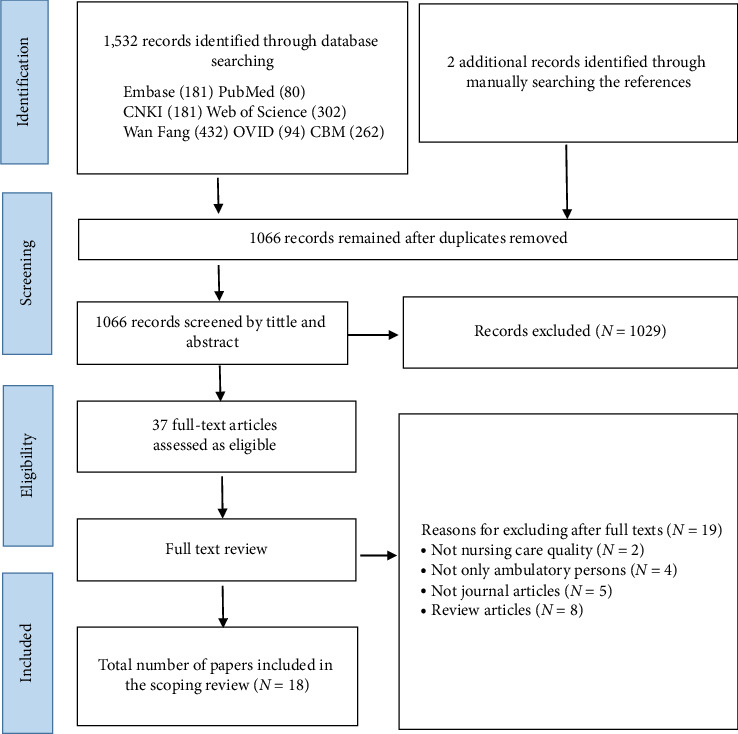
PRISMA flow diagram of included studies.

The database search identified a total of 1532 articles: 181 from Embase, 80 from PubMed, 302 from Web of Science, 94 from OVID, 181 from CNKI, 432 from Wan Fang, and 262 from CBM. In addition to this, two additional records were found through manual reference searching. After removing 468 duplicates, two authors independently screened titles and abstracts (*n* = 1066) to exclude studies that did not meet the inclusion criteria. Subsequently, the remaining 37 studies were reviewed for full text, with a further 19 irrelevant researches being eliminated. Finally, after thorough review and screening processes, a total of 18 papers met the eligibility criteria and were included in the final synthesis. The PRISMA flowchart is shown in Figure 2.

### 4.2. Study Characteristics Summary

**Figure 3:**
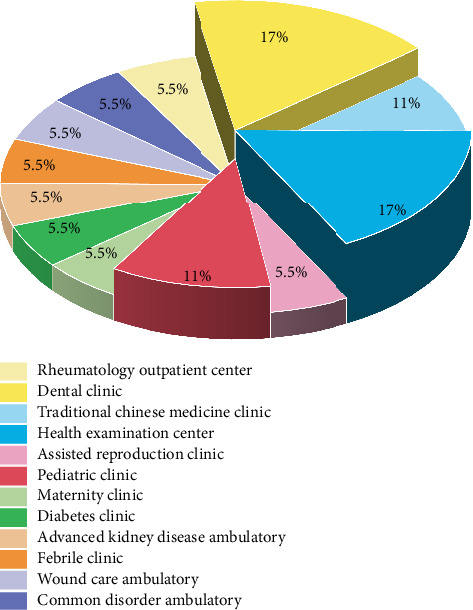
The 3D pie chart of outpatient type distribution.

**Figure 4:**
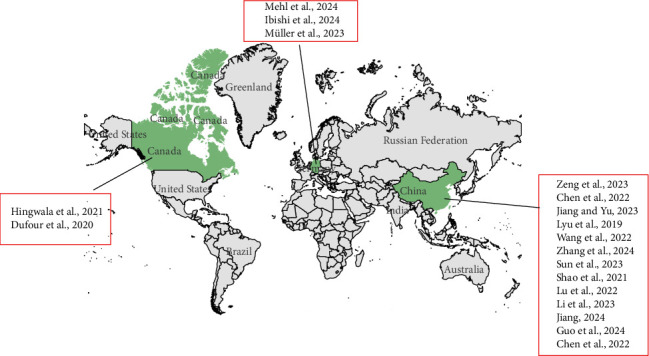
The regional distribution map.

Table 1 provides a summary of the included studies. Of the 18 studies, one were quantitative [47] and 17 were mixed methodology [37–46, 48–53]. Among the 17 mixed studies, 13 utilized literature analysis for data collection [37, 39–41, 43–46, 48–50, 52, 54], seven employed semistructured interviews [38, 40, 41, 46, 48, 50] and 13 utilized Delphi expert consultation [39–46, 48, 50–52]. The 18 studies covered a wide range of outpatient types. Three studies were conducted in the dental clinic, two in the traditional Chinese medicine clinic, two in the pediatric clinic, and three in the health examination center. Additionally, there was one study each conducted in the rheumatology outpatient center, common disorder ambulatory, assisted reproduction clinic, diabetes clinic, advanced kidney diseases ambulatory, febrile clinic, and wound care ambulatory. The 3D pie chart isdepicted in Figure 3. The included studies were conducted in different countries, with two being undertaken in Canada, three in Germany and 13 in China. A world map, which delineates the names of track and trigger charts and study locations, is included in Figure 4.

### 4.3. Summary of Outpatient Care Quality Indicators

#### 4.3.1. Structure

Table 2 shows the summary of quality indicators of ambulatory care based on Donabedian model. According to Donabedian's three-dimensional quality model, the quality structure indicators of outpatient care mainly consist of four secondary indicators. These include outpatient management system and norms, organizational environment, nursing human resources, and material resource allocation. The outpatient management system and norms mainly focus on eight tertiary indicators: outpatient nursing management process, system and responsibilities, service evaluation mechanism, emergency response plan, nurse access system, fee project management system, and technology access standards. The organizational environment primarily considers the presence of visible signage, independent consulting rooms, and adherence to outpatient infection management requirements within the clinic. Nursing human resources mainly evaluate the ratio of doctors/nurses, the proportion of nurses with different degrees, the proportion of nurses with different professional titles, the proportion of nurses with different working years, the turnover rate, the composition of nursing staff levels and the flexible deployment of human resources in emergency situations. The material resources mainly consist of four tertiary indicators: the reasonable allocation of materials and equipment, clear labeling, fixed position, and equipment availability.

#### 4.3.2. Process

The process indicators of outpatient nursing quality mainly involve nine secondary indicators of outpatient specialty nursing practice, medical management, nursing safety, health education, patient identification, nursing records, infection control, material, and equipment management and humanistic care. Specialized nursing practice mainly assesses the implementation of outpatient nursing training and the pass rate, the pass rate of first aid skills and the pass rate of professional skills. Medical management mainly includes 3 tertiary indicators of patient waiting time, treatment duration and triage. Nursing care safety includes the correct use of warning signs, identification and management of common outpatient emergencies (blood sickness, needle sickness, hypoglycemia, syncope, cardiac arrest, and etc.), implementation of fall protection and implementation rate of risk assessment for high-risk patients. Infection control is an important element of outpatient care quality indicators, including five tertiary indicators of standardized prevention, hand hygiene, occupational exposure, contact surface disinfection and standardized rates of medical waste collection and disposal. Humanistic care is mainly involved in 5 tertiary indicators: convenience measures (paper towels, paper cups, water dispensers, self-service vending machines, and etc.), communication and coordination skills of outpatient nurses, implementation rate of privacy protection, implementation rate of patients' and family members' needs assessment, and implementation rate of patients' and family members' emotional calming. Please refer to Table 2 for more detailed information.

#### 4.3.3. Outcome

The outcome section primarily assessed four secondary indicators: satisfaction, nursing adverse events, high quality nursing, and outputs. Satisfaction includes three tertiary indicators: patient satisfaction with nursing, doctor satisfaction with outpatient nurses, patient complaint incidence and effective handling rate. Adverse nursing events were evaluated using five tertiary indicators: the occurrence of falls, swallowing and aspiration incidents, pressure ulcers, nursing errors, and occupational exposures. In addition to this, the development of new technologies and programs in nursing, the number of patents filed and papers published, and the number of patients seen were used as tertiary indicators to assess the quality of care in the outpatient clinics. Details are provided in Table 2.

## 5. Discussion

Previous studies have shown that the application of the Donabedian model in the quality of care has shown high-quality development [55, 56], breaking the subjective evaluation of quality evaluation, to a certain extent, promoting the improvement of the evaluation of the quality of care, and using this to push the quality improvement and evaluation to a more scientific and standardized category. The model has been widely used and evaluated in emergency departments, operating rooms, intensive care units, and oncology departments [19–21], but its use in outpatient settings began late and remains limited. The 18 articles included in this study show that the quality of outpatient care has gradually begun to receive attention in recent years, and all are exploring outpatient-specific quality of care indicators to more scientifically assess the quality of outpatient care. However, the shortcoming lies in the underutilization of the Donabedian model in outpatient clinics, as evidenced by its limited application and insufficient research papers on the subject. We anticipate that the Donabedian model will be further developed and expanded to encompass the quality of outpatient care.

This study involved oral, fever, traditional Chinese medicine, advanced kidney disease, and pediatric outpatient care. Each outpatient population has its own unique characteristics. We reviewed 18 papers and found that quality of care indicators constructed based on the structure-process-outcome model have commonalities as well as their own peculiarities. In the structure, institutional norms and environmental settings are emphasized in all specialty clinics. During the process, each specialty clinic incorporated an assessment of specialty-adapted technologies based on its own characteristics. For example, Chinese medicine outpatient clinics assessed the correct rate of implementation of evidence-based care, the standardization rate of Chinese medicine nursing techniques, and the implementation rate of Chinese medicine health promotion in the nursing process [40]. Due to the unique characteristics of pediatric outpatient clinics [57], such as the young age of patients and their lack of behavioral control, quality assessment of nursing processes in these clinics focuses on standardized venous blood collection, use of restraining devices, psychological assessment of children, proper disposal procedures, effective communication among family members, and education for family members [44]. These differences in specialty characteristics lead to the need for further validation and harmonization of outpatient care quality indicators constructed on the basis of the Donabedian model. In addition to this, the process indicators of infection prevention and nursing safety are all key sensitive indicators of nursing quality that have gained wide international recognition [3]. Among the outcomes, satisfaction and adverse events of care were the key indicators to assess the quality of care. Satisfaction assessment is important in improving the patient experience, building healthy nurse-patient relationships, and promoting continuous improvement in nurse-patient services [58]. The importance of assessing nursing adverse events is to promptly identify and address issues in the nursing process, prevent recurrence of similar events, enhance the quality of nursing care, ensure patient safety, support professional development of nursing staff, and create a safer healthcare environment [59].

Reviewing these 18 studies conducted in outpatient clinics, we found that researchers mostly used mixed studies when applying the Donabedian model. Mixed studies often use Delphi, semistructured interviews, hierarchical analysis, or evidence-based methods to construct a quality evaluation system for each outpatient specialty and sensitive indicators of quality of care to assess the quality of outpatient care. Although there are lots of studies that use the model to construct the evaluation system, few scholars have applied the constructed evaluation system. Only one of the included studies [44] applied the constructed evaluation system clinically and evaluated the effect. In addition, there was only one quantitative study among 18 studies [47], which constructed a continuum of care intervention based on the structure-process-outcome model to evaluate the effectiveness of the intervention for patients with gestational diabetes mellitus (GDM). Quantitative studies mainly use the model as a basis for constructing more optimized care plans, aiming to provide better quality care for patients. It is worth noting that despite the uniformity of the research methodology used in the mixed study, there were differences in the results of the study due to the specific attributes of each outpatient specialty. The number of indicators at each level, the importance of each indicator, and the weighting of each indicator are different in the results of each outpatient specialty, which may lead to limitations in the uniformity and application of outpatient nursing care quality evaluation standards, affecting the extrapolation of the research results and their clinical application. In the future we expect more extensions of the Donabedian model in outpatient quality of care studies. For example, conducting more intervention studies with large samples based on structure-process-outcome models to optimize more care protocols. Qualitative research is also the next hot topic that can be explored in this field.

### 5.1. Limitation

This scoping review provides an overview of published research on the quality of outpatient care based on the Donabedian model over the last 5 years. It uses a systematic search strategy as transparent and easily repeatable as a systematic evaluation [60]. However, it is still important to point out some limitations of this study. The first limitation is that this study did not search all relevant databases.

To expand the search and increase results yield the search could be repeated in other databases such as MEDLINE, CINAHL, the Joanna Briggs Institute, and Google Scholar. It could have also included nonresearch publications. This review focus on the application of the Donabedian model to the quality of outpatient care, rather than the systematic construction of quality indicators for outpatient care. This may be a limitation for scholars who are interested in exploring the construction of metrics for outpatient care. A third potential limitation is that the subject of the study was an outpatient clinic, which may limit generalizability. However, the purpose of this review is to provide an overview of published works related to the topic to inform the reader and help identify the need for further research on the topic, which has been accomplished.

## 6. Conclusion

This study provides an overview of the application of the Donabedian model in outpatient care quality, including study types, outpatient types, regional distribution, and summarizes outpatient care quality indicators from the structure-process-outcome. Outpatient care quality studies based on the Donabedian model are predominantly mixed, with a relatively homogenous type of study. Mixed studies have used data analysis, Delphi method, and semistructured interviews to innovate and improve the evaluation indexes of outpatient specialty care quality. However, these are only constructing the index system, not applying it in practice, or only focusing on single-center and small-sample applications, lacking the validation of large sample sizes in clinical practice. Interventional and qualitative research would be the next frontier. Multicenter and large sample applications are needed in the future to make it a better value in nursing practice to promote the continuous improvement of outpatient nursing quality. Structural indicators of outpatient care quality provide additional policy and resource support for nursing managers. Process quality involves different outpatient clinic types, and specialty nursing practice indicators have variability and still require further validation and harmonization. Satisfaction and adverse events are core components of outpatient care quality outcome evaluation and important for continuous quality improvement.

## 7. Implications for Nursing Management

The application of the Donabedian model including study types, outpatient types, regional distribution, and quality indicators, as summarized in this review, offers novel insights and references for nursing managers aiming to enhance the quality of outpatient nursing care. Nursing administrators should actively emphasize and effectively assess important quality indicators such as adverse events, satisfaction, and safety of care, and place outpatient safety, satisfaction, and quality improvement at the forefront of improving the quality of outpatient care. In addition, nursing managers should explore more nursing quality practice methods based on the Donabedian model, listen to patients' voices on nursing quality, and apply qualitative research and intervention methods to quality management.

## Figures and Tables

**Table 1:** General characteristics of selected studies.

Author, year	Country	Objective	Outpatient institution	Research methodology	Quality model	Findings
Mehl et al. [37]	Germany	To develop an indicator set for the evaluation of the quality of routine ambulatory healthcare for common disorders in children and adolescents	Ambulatory health care for asthma, atopic eczema, otitis media, tonsillitis, attention-deficit hyperactivity disorder (ADHD), depression and conduct disorder	Literature analysis method, modified RAND-UCLA appropriateness method (RAM)	Donabedian and OECD quality standard (effectiveness, safety, patient-centered)	89% of the indicators capture process quality, 9% outcome quality and 2% structural quality; according to the OECD classification, 61% measure effectiveness, 23% patient-centeredness and 16% safety of care
Ibishi et al. [38]	Germany	To assess patient satisfaction, needs, expectations and preferences for care and to formulate recommendations to improve the quality of care in a large tertiary rheumatology center	Rheumatology outpatient center	Semistructured interview, content-structuring qualitative content analysis	Donabedian model	6 recommendations for structural quality optimization; 7 recommendations for process quality optimization and 1 recommendation for outcome quality optimization, specifically to optimize personnel management, internal practice processes, practice equipment and treatment processes in the outpatient clinic
Zeng et al. [39]	China	To construct sensitive quality indicators for dental outpatient care	Dental outpatient	Literature analysis method, Delphi expert consultation	Donabedian (structure-process-outcome) model	3 structural quality indicators, 7 process quality indicators, 19 outcome quality indicators
Chen et al. [40]	China	To construct an evaluation index system for the quality of traditional Chinese medicine outpatient nursing care and providing a tool for evaluating the quality of nursing care	Traditional Chinese medicine (TCM) clinic	Literature analysis method, semistructured interview, Delphi expert consultation	Donabedian model	3 structural quality indicators, 5 process quality indicators, 53 outcome quality indicators
Jiang and Yu [41]	China	To construct a sensitive indicator system for quality of care in health examination centers	Health examination center	Literature analysis method, interview, Delphi expert consultation	Donabedian (structure-process-outcome) model	3 structural quality indicators, 11 process quality indicators, 38 outcome quality indicators
Lyu et al. [42]	China	To explore the evaluation index of nursing quality of assisted reproduction center, and provide the basis for the establishment of nursing management evaluation system of assisted reproduction center	Assisted reproduction clinic	Brainstorming, Delphi expert consultation, questionnaire methods	Structure-process-outcome	16 quality of care indicators, including 2 structural indicators, 9 process indicators and 5 outcome indicators
Wang et al. [43]	China	To construct sensitive quality indicators for monitoring and evaluating the quality of dental clinic	Dental clinic	Literature analysis method, interview, Delphi expert consultation	“Structure-process-outcome” three-dimensional model	3 structural quality indicators, 10 process quality indicators, 26 outcome quality indicators
Zhang et al. [44]	China	To construct the evaluation index system of the quality of pediatric outpatient nursing service, and explore the application effect	Pediatric clinic	Literature analysis, Delphi expert correspondence, randomized controlled intervention, questionnaire approach	“Structure-process-outcome” three-dimensional model	3 structural quality indicators, 9 process quality indicators, 43 outcome quality indicators The indicators can help to improve the quality of nursing services and enhance the satisfaction of nursing care of children's families
Sun et al. [45]	China	To construct indicators for evaluating the quality of Chinese medicine outpatient nursing care and provide a basis for systematic, scientific and objective monitoring and evaluation of the quality	Traditional Chinese medicine (TCM) clinic	Literature analysis, Delphi expert correspondence	Donabedian model	3 structural quality indicators, 10 process quality indicators, 42 outcome quality indicators
Shao et al. [46]	China	To construct a sensitive nursing quality index system applicable to geriatric dental outpatient clinics to provide a standardized and quantifiable basis for strengthening nursing quality management and safeguarding the safety of geriatric patients	Geriatric stomatological outpatient	Literature analysis, semistructured interview, Delphi expert correspondence, analytic hierarchy process (AHP)	“Structure-process-outcome” three-dimensional quality model	3 structure indicators, 12 process indicators and 58 outcome indicators
Lu et al. [47]	China	To study the effect of continuous nursing interventions based on structure-process-outcome model in patients with gestational diabetes mellitus (GDM)	Maternity clinic	Randomized controlled intervention, questionnaire method	“Structure-process-outcome” three-dimensional quality model	Glucose metabolism index, self-management ability and quality of life were improved, and pregnancy outcome was good
Li et al. [48]	China	To construct the quality evaluation index system of diabetes nurse-led clinic	Diabetes nurse-led clinic	Literature analysis, semistructured interview, Delphi expert correspondence, analytic hierarchy process (AHP)	“Structure-process-outcome” three-dimensional quality model	3 structure indicators, 11process indicators and 69 outcome indicators
Mueller et al. [49]	Germany	To examine the scope, quality dimensions and treatment aspects covered by existing quality indicators (QIs) for the somatic diseases	Pediatric clinic for seven common diseases	Literature analysis method, generalization	Donabedian and OECD quality standard (effectiveness, safety, patient-centered)	78% focused on process quality, 20% on outcome quality and 2% on structural quality; According to OECD criteria, 72% of the indicators were assigned to effectiveness, 17% to patient-centeredness, 11% to patient safety and 1% to efficiency
Jiang [50]	China	To construct a sensitive indicator system for quality of care in medical examination centers	Medical examination centers	Literature analysis, semistructured interview, Delphi expert correspondence	Donabedian model	3 structure indicators, 11 process indicators and 38 outcome indicators
Hingwala et al. [51]	Canada	To identify, categorize, and evaluate quality indicators for ambulatory patients with advanced kidney disease	Ambulatory for advanced kidney disease	Indicator collection, indicator evaluation, modified Delphi method	The institute of medicine and Donabedian frameworks	None of the indicators rated as necessary measured timely or equitable care, nor did they identify any measures that assessed the setting in which care occurs (i.e., structure measures)
Guo et al. [52]	China	To establish of a set of scientific, sensitive, actionable and representative quality-sensitive indicators for febrile outpatient care	Febrile outpatient care	Literature analysis, Delphi expert correspondence	Donabedian model	3 structure indicators and 19 process indicators
Dufour et al. [53]	Canada	To measure primary care nursing indicators using a wound care tracer condition and explore the associations between process and outcome indicators	Wound care ambulatory	Descriptive analyses (bivariate analyses, Pearsonχ2testfor homogeneity)	Process-outcome model	Continuity and follow-up of care are associated with improved outcomes, and increasing the level of performance of these indicators can improve both care processes and patient outcomes
Chen et al. [40]	China	To construct a quality-of-care evaluation index system for health management centers	Health management centre	Literature analysis, semistructured interview, Delphi expert correspondence, analytic hierarchy process (AHP)	Structure-process-outcome model	3 structure indicators, 11process indicators and 41 outcome indicators

**Table 2:** Summary of quality indicators of ambulatory care based on the Donabedian model.

Primary indicators	Secondary indicators	Tertiary indicators
Structure	Outpatient management system and norms	Clear outpatient care management process
		Clear outpatient nursing management system
		Clear ambulatory nursing position responsibilities
		Complete outpatient nursing service evaluation mechanism
		Emergency response plan for emergencies
		Nurse admittance system
		Charge item management system
		Outpatient nursing technology access system
	Organizational environment	Visible signage
		Separate consultation rooms
		Compliance with outpatient infection management requirements
		Complete safety equipment (e.g., handrails, alarms)
	Nursing manpower allocation	Doctor/nurse ratio
		The proportion of nurses with different education background
		The proportion of nurses with different professional titles
		The proportion of nurses with different working years
		Quit rate
		Nursing staff level composition
		Emergency flexible deployment of human resources
	Allocation of material resources	Reasonable configuration of materials and equipment
		Clearly labelled and placed in a fixed location
		Instruments and equipment intact rate

Process	Outpatient specialist nursing practice	Nursing training and assessment implementation and pass rate
		First aid skills pass rate
		Specialist skills pass rate
	Medical treatment management	Waiting time for patients to be seen
		Waiting time for medical treatment
		Triage
	Nursing safety	Correct use of warning signs
		Recognition and management of common outpatient emergencies (blood and needle sickness, hypoglycemia, syncope, cardiac arrest, etc.)
		Fall protection implementation
		Execution rate of risk assessment for high-risk patients
	Health education	Health education coverage and awareness of outpatient diseases
	Patient identification	Accurate matching of identity information
	Nursing record	Outpatient care and information records (mental health, quality of life for patients, and families)
	Infection control	Standardized prevention
		Hand hygiene
		Occupational exposure
		Disinfection of contact surfaces
		Medical waste collection and disposal standardization rate
	Material and equipment management	Qualified rate of standardized management of high value consumables
		First-aid equipment and medicine readiness rate
	Humanistic care	Convenience measures (paper towels, paper cups, water dispensers, self-service vending machines, etc.)
		Outpatient nurses' communication and co-ordination skills
		Privacy protection implementation rate
		Implementation rate of patient and family needs assessment
		Implementation rate of patient and family emotional calming

Outcome	Satisfaction	Patient satisfaction
		Doctor satisfaction
		Patient complaint incidence and effective handling rate
	Nursing adverse events	Incidence of falls
		Incidence of swallowing and aspiration
		Incidence of pressure injury
		Incidence of medication administration errors by nursing staff
		Incidence of occupational exposure of nursing staff
	Quality nursing care	Outpatient appointment
		Outpatient follow-up
	Outputs	The development of new technologies and project in nursing
		Number of patent applications and papers published
		Number of patients

## Data Availability

The data that support the findings of this study are available from the corresponding author upon reasonable request.

## Supporting Information

Table S1: Search terms for various databases. Please note: The publisher is not responsible for the content or functionality of any supporting information supplied by the authors. Any queries (other than missing content) should be directed to the corresponding author for the article.
