# Supplementary material for: Application of Donabedian Three-Dimensional Model in Outpatient Care Quality: A Scoping Review
Source: J Nurs Manag. 2025 Apr 24;2025:6893336. doi: 10.1155/jonm/6893336 (PMC12045680; doi:10.1155/jonm/6893336)
Supplement: Supporting Information — Additional supporting information can be found online in the Supporting Information section. [file 6893336.f1.docx]

| Table S1. Search terms for various databases | | |
| --- | --- | --- |
| Database | Search strategy | Records |
| PubMed | **(((((("Outcome Process Assessment"[Title/Abstract]) OR (Donabedian Trial[Title/Abstract])) OR (Structure Process Outcome Triad[Title/Abstract])) OR (Donabedian Model[Title/Abstract])) OR (Triad, Donabedian[Title/Abstract])) OR (Model, Donabedian[Title/Abstract])) AND ((((((((Quality of Health Care[MeSH Terms]) OR (Quality Indicators, Health Care[MeSH Terms])) OR (Health Care Quality[Title/Abstract])) OR (Quality of Care[Title/Abstract])) OR (Care Quality[Title/Abstract])) OR (Quality of Healthcare[Title/Abstract])) OR (Healthcare Quality[Title/Abstract])) OR (Quality Indicator*[Title/Abstract]))** | 80 |
| Web of science | (((TS=(“Outcome Process Assessment”) OR TS= (“Donabedian Trial”) OR TS= (“Structure Process Outcome Triad”) OR TS= (“Donabedian Model”) OR TS=(Donabedian)) AND (TS=(“Quality of Health Care”) OR TS=(“Quality Indicator*”) OR TS=(“Health Care Quality”) OR TS=(“Quality of Care”) OR TS=(“Care Quality”) OR TS=(“Quality of Healthcare”) OR TS=(“Healthcare Quality”))) AND PY= (2019-2024)) | 302 |
| Embase | (('outcome process assessment':ab,ti OR 'donabedian trial':ab,ti OR 'donabedian':ab,ti OR 'structure process outcome':ab,ti OR 'structure process outcome triad':ab,ti OR 'donabedian model':ab,ti) AND ('quality of health care':ab,ti OR 'quality indicator*':ab,ti OR 'health care quality':ab,ti OR 'quality of care':ab,ti OR 'care quality':ab,ti OR 'quality of healthcare':ab,ti OR 'healthcare quality':ab,ti)) | 181 |
| OVID | ((Outcome Process Assessment.ab,kw,ti. or Donabedian Trial.ab,kw,ti. or Donabedian.ab,kw,ti. or Structure Process Outcome Triad.ab,kw,ti. or Structure Process Outcome.ab,kw,ti. or View results by resource  Donabedian Model.ab,kw,ti.) and (Quality of Health Care.ab,kw,ti. or  "Quality Indicator*".ab,kw,ti. or Health Care Quality.ab,kw,ti. or Health Care Quality.ab,kw,ti. or Quality of Care.ab,kw,ti. or Care Quality.ab, kw,ti. or Quality of Healthcare.ab,kw,ti. or Healthcare Quality.ab,kw,ti.)) | 94 |
| CNKI | (SU %= 'Structure-Process-Outcome'+'Three-dimensional Model'+'Donabedian' OR KY = 'Structure-Process-Outcome'+'Three-dimensional Model'+'Donabedian' OR TI = 'Structure-Process-Outcome'+'Three-dimensional Model '+'Donabedian') AND (SU %='Nursing service quality'+'nursing quality'+'nursing work quality '+'medical care quality'+'nursing safety'+'medical service quality'+'nursing effect'+'nursing quality management' OR KY = 'Nursing service quality'+'nursing quality'+'nursing work quality '+'medical care quality'+'nursing safety'+'medical service quality'+'nursing effect'+'nursing quality management' OR TI = 'Nursing service quality'+'nursing quality'+'nursing work quality '+'medical care quality'+'nursing safety'+'medical service quality'+'nursing effect'+'nursing quality management') | 181 |
| Wan Fang | (Theme:("Structure-Process-Outcome" or "Three-dimensional Model" or "Donabedian") or Tittle or key words:( "Structure-Process-Outcome" or "Three-dimensional Model" or "Donabedian")) and (Theme:( "Nursing service quality" or "nursing quality" or "nursing work quality" or "medical care quality" or "nursing safety" or "medical service quality" or "nursing effect" or "nursing quality management") or Tittle or key words:(" nursing service quality" or "nursing quality" or "Quality of nursing work" or "quality of medical care" or "Safety of nursing" or "quality of medical service" or "Effect of nursing" or "management of nursing quality")) | 432 |
| CBM | ("Structure-Process-Outcome " [core field: intelligent] OR "Three-dimensional Model" [core field: intelligent] OR "Donabedian"[Common field: intelligent])) AND ("Nursing service quality"[core field: intelligent] OR "nursing quality"[core field: intelligent] OR "nursing work quality"[core field: intelligent] OR "medical care quality"[core field: intelligent] OR "nursing safety"[core field: intelligent] OR "medical service quality"[core field: intelligent] OR "nursing effect"[core field: intelligent] OR "nursing quality management"[core field: intelligent] ) | 262 |
| Note. TS=topic; ab=abstract; TI=tittle; kw=key words; SU=subject; KY=key words | | |
